# Patterns of divergence in fish species separated by the Isthmus of Panama

**DOI:** 10.1186/s12862-017-0957-4

**Published:** 2017-05-10

**Authors:** Christine E. Thacker

**Affiliations:** Research and Collections, Section of Ichthyology, Natural History Museum of Los Angeles County, 900 Exposition Blvd, Los Angeles, CA 90007 USA

**Keywords:** Isthmus of Panama, Divergence time extimates, Rate shifts

## Abstract

**Background:**

The Pleistocene closure of Isthmus of Panama, separating the basins of the Eastern Pacific and the Caribbean Sea, created a unique natural experiment that reveals how marine faunas respond to environmental change. To explore how fishes have been affected by this tectonic event, I compare transisthmian patterns in phylogeny and morphology for geminate lineages in two families, Eleotridae (sleepers) and Apogonidae (cardinalfishes).

**Results:**

Time-calibrated phylogenies for these families show different diversification patterns. In Eleotridae, several independent shallow instances of transisthmian divergences occur, with one or a few species on either side of the Isthmus. Among Apogonidae, a single clade of Eastern Pacific species is nested within a broad Caribbean radiation that also includes a species known from the Mediterranean. Divergence time estimates for taxa isolated by closure of the Isthmus are broadly congruent. Hypotheses dated with deeper, fossil-based legacy calibrations put the divergences in the Miocene at 7.4–15.1 Ma, while those estimated with a shallow biogeographic calibration of final Isthmus closure range from 5.1 to 9.9 Ma, in the late Miocene/early Pliocene. Eleotridae are more euryhaline than Apogonidae, but do not exhibit shallower transisthmian divergences. In both families, descendent lineages on either side of the Isthmus of Panama exhibit significant shape differences, although that distinction disappears for Apogonidae when I apply a correction for phylogenetic relationships. To evaluate the tempo and mode of continuous character evolution, I fit several single and multiple rate evolutionary models to morphometric data reconstructed on the Apogonidae phylogeny. I find that the most highly favored model, as estimated on both legacy and isthmus calibrated hypotheses, is a multiple rate Ornstein-Uhlbeck model, with a mosaic of rate shifts postulated for shape changes among fishes in the Caribbean and Eastern Pacific.

**Conclusions:**

Although many transisthmian taxa have been compared and their phylogenies calibrated to estimate the dates associated with population sundering, few studies correlate these timing estimates with morphological change. I show that in transisthmian fish lineages, morphometric distinctions are detectable across the Isthmus, and that rates and patterns of shape change have also shifted, with variable manifestations across the body and between the Caribbean and Eastern Pacific.

**Electronic supplementary material:**

The online version of this article (doi:10.1186/s12862-017-0957-4) contains supplementary material, which is available to authorized users.

## Background

Complex interaction between organismal morphology and environment yields the tremendous scope of variation displayed across the tree of life. Underlain by genetics and mediated by selection, evolutionary changes in response to environmental conditions are at the core of comparative biology. Examining and understanding how morphology covaries with environment requires comparison of species in different habitats and evaluation of their similarities and differences. In marine areas, organismal ranges are often wide, with diffuse boundaries, making finer-scale contrasts difficult. However, the world ocean is divisible into broad regions, each with distinct environments and faunas. Comparisons between these regions can inform understanding of broad-scale historical and evolutionary patterns in marine organisms. A prime example of neighboring marine environments separated relatively recently into disparate ecotopes is the division of the Eastern Pacific from the Caribbean Sea by the closure of the Isthmus of Panama.

The Isthmus of Panama is one of the most familiar and well-studied examples of a land barrier separating marine environments. Linking North and South America, this land bridge sundered the Eastern Pacific from the Caribbean Sea gradually over a period of 12 million years, finally closing completely in the Pleistocene, three to four million years ago (Ma; [1]). Establishment of this land bridge not only split marine populations, it also altered their habitats. After the closure, tropical currents in the Caribbean no longer flowed to the west and were instead deflected northward, causing increases in both salinity and temperature, and strengthening the Gulf Stream in the Atlantic. The Eastern Pacific, sealed off from its input of tropical ocean waters, cooled and became nutrient-rich due to deep water upwelling off the Central American coast [2–4]. These dramatic ecological changes likely resulted in extinctions in both oceans, and left behind geminate lineages that then further diverged.

The genetic signature of Isthmus closure is detectable throughout transisthmian clades, although interestingly the divergence dates for geminate lineages vary. Among the marine organisms surveyed, a minority have inferred split dates at (or sometimes after) the final Isthmus closure. More commonly, the genetic pattern is consistent with population separation in the 10 million years prior to final closure, during the gradual uplift and assembly of the Isthmus in the late Miocene [3–5]. Although many studies have considered the genetic divergence of geminate transisthmian taxa, few have examined the phenotypic consequences of the vicariance and ecological shifts precipitated by Isthmus closure. Previous work on the mojarra genus *Diapterus* [6] indicated that species separated by the Isthmus of Panama were morphometrically separable in some but not all cases, and analysis of size evolution across the Isthmus among snooks (*Centropomus*) did not reveal a consistent transisthmian pattern [7]. Outside fishes, geminate arcid bivalve species are not completely separated in morphospace, but are morphometrically distinguishable in most (84%) cases [8]. Environmental changes following closure of the Isthmus affected the entire marine fauna, naturally assembling a wide-scale marine evolutionary experiment, but the responses of marine taxa to these tectonic changes have been gradual and complex.

The aims of this study are to examine representatives of lineages separated by the Isthmus of Panama, to evaluate when and in what ways they have diverged, and to determine whether or not divergence rates or patterns changed following the uplift of the Isthmus. I selected taxa from two acanthomorph fish families, Eleotridae and Apogonidae, both in the order Gobiiformes. Eleotridae (sleepers) are generally euryhaline, inhabiting brackish and freshwaters as well as nearshore marine environments; Apogonidae (cardinalfishes) are fully marine and associated with nearshore benthic habitats. For Eleotridae I focused on geminate pairs in the genera *Dormitator*, *Eleotris*, *Gobiomorus,* and *Leptophilypnus*. These genera are phylogenetically distinct and represent all of the three New World eleotrid clades [9]. Apogonidae manifest a different phylogenetic pattern, with a single primarily New World clade including *Apogon* species, as well as all members of the genera *Astrapogon*, *Phaeoptyx*, *Paroncheilus* and *Zapogon*. The New World Apogonidae clade is composed of a broad Caribbean radiation with an Eastern Pacific clade nested within it, and including a single species that inhabits the Mediterranean Sea [10, 11]. For these taxa, I sampled throughout the New World clade, including the Mediterranean species.

I used an integrated morphological and molecular approach to evaluate transisthmian evolutionary patterns among Apogonidae and Eleotridae. For each group, I assembled and calibrated a phylogeny, in order to date the transisthmian splits for each geminate pair as well provide an evolutionary framework for the pattern of morphological change. To investigate how calibration can affect the inferred dates, as well as subsequent comparative analyses of shape change, I calibrated the phylogenies in two ways. First, I used divergence dates for deep nodes in the tree, derived from previous fossil-calibrated phylogenies (legacy calibration). In a second analysis, I calibrated shallow nodes of transisthmian relatives with a minimum date of 3.1 Ma, the time of the final closure of the Isthmus of Panama (isthmus calibration).

To assess change in form among species and between regions, I used geometric morphometrics. I digitized a suite of 17 external morphological landmarks among transisthmian relatives to measure shape variation and gauge whether and in what ways the transisthmian lineages have diverged. For nonindependent (phylogenetically nested) transisthmian clades, I applied phylogenetic corrections to the morphometric data to account for common ancestry. I also applied and compared single and multiple rate models to the morphometric characters reconstructed on the calibrated phylogenies, to ascertain whether or not rates or modes of phenotypic change shifted following closure of the Isthmus of Panama and resultant environmental changes in both ocean basins. I performed all the phylogenetically corrected analyses on both the legacy calibrated and the isthmus calibrated hypotheses, to evaluate the effect of the different calibration regimes.

## Methods

### Phylogenetic reconstruction and calibration

To infer phylogeny for Eleotridae and Apogonidae, I assembled previously published DNA sequence data and used Bayesian methods for both phylogenetic analysis and time-calibration with fossil-based legacy dates. For Eleotridae I used the matrix of [9], including Rhyacichthyidae, Odontobutidae, Milyeringidae, Butidae, and Eleotridae. The dataset includes sequence from the mitochondrial genes cytb, COI, ND1, and ND2, totaling 4397 base pairs for 78 taxa. That phylogeny [9] inferred that there are three clades of New World taxa within Eleotridae, of which two are sisters, consistent with two separate invasions of New World waters. Resolution among the major clades within the family was weakly supported. For this study I have included a wide range of gobiiform taxa, both to provide phylogenetic context for the New World taxa as well as to permit assignment of fossil calibrations to the tree. I also provide a Bayesian analysis of the [9] dataset, which was originally analyzed with parsimony. Eleotrid genera containing or entirely consisting of New World species are *Dormitator*, *Eleotris*, *Gobiomorus*, *Guavina*, *Hemieleotris*, *Leptophilypnus* and *Microphilypnus*. I use the genus name *Eleotris* for *E. armiger* and *E. smaragdus*, species often placed in the genus *Erotelis*, in line with the phylogenetic evidence indicating that *Erotelis* is nested within *Eleotris* [9, 12, 13]. Of the New World eleotrid genera, all except for *Microphilypnus* (known from Venezuela and Brazil) have representatives on both sides of the Isthmus of Panama. For this study, all genera are present in the phylogeny, but representatives of transisthmian geminate pairs were only available for *Dormitator*, *Eleotris*, *Gobiomorus*, and *Leptophilypnus*. Sequences used for phylogenetic analysis of Eleotridae are given in Additional file 1: Table S1.

For Apogonidae, I analyzed a subset of the matrix of [10], including both mitochondrial (COI) and nuclear (ENC1, RAG1) genes, for a total of 3600 aligned base pairs for 26 taxa. Previous phylogenies of Apogonidae have varied in the depth of their sampling, but all agree in grouping New World and Mediterranean *Apogon* with *Astrapogon*, *Paroncheilus*, *Phaeoptyx*, and *Zapogon*, as part of a fairly deep split in the apogonid tree [10, 11, 14]. For this analysis, I included all the *Apogon*, *Astrapogon*, *Paroncheilus*, *Phaeoptyx*, and *Zapogon* sequenced by [10], as well as *Gymnapogon vanderbilti* and *Pseudamia gelatinosa*, used as outgroups and to provide deep enough nodes for calibrating the hypothesis. Sequences used for phylogenetic analysis of Apogonidae are listed in Additional file 1: Table S2.

I assembled the matrices using Geneious version 6.1.8 and Mesquite version 3.0.4, and aligned the protein coding genes based on the translated amino acid sequence. I then used MrBayes, version 2.0.9 to infer the Bayesian phylogeny (implemented in Geneious). For that analysis, I specified models independently for each gene partition. I applied a HKY + G + I substitution model for the COI partition and a GTR + G + I model, independently, for each of the cytb, ND1, and ND2 gene partitions in Eleotridae. Similarly, I used the GTR + G + I model for each of the COI and RAG1 gene partitions and SYM + G + I for the ENC1 gene partition in Apogonidae (as chosen by the R module *phangorn* [15]). I ran the analysis for 10.0 × 10^7^ generations, with four simultaneous chains, sampling every 1000 replications, and discarding the first 10% of trees as burn-in. I constructed a 50% majority-rule consensus phylogeny of the remaining trees, then calibrated that phylogeny with Beast 1.7.5 [16], run with an uncorrelated lognormal relaxed clock model and a birth/death speciation prior.

To assign calibrations to the hypotheses, I first used dates inferred based on fossil calibrations in previous, more deeply sampled phylogenies. The use of such secondary calibration points has been questioned, particularly when they are assigned without error [17, 18], and one study showed that assignment of secondary calibrations results in small (1–2 Ma) but significant differences among subsamples of simulated phylogenies [19]. For these data, another possible method of calibration is to assign the node subtending the least divergent transisthmian lineages a biogeographic calibration, based on the date of the closure of the Isthmus of Panama. The use of biogeographic calibrations has also been criticized [20–22], in particular the assumptions that the date of a biogeographic event is reliably known and that no extinction has occurred since then that could result in the calibration being applied erroneously. Both calibration methods have their drawbacks, but because it is instructive to compare them, I performed a second calibration analysis for each family, with the shallowest (or only) transisthmian lineage in each group assigned a minimum divergence of 3.1 Ma.

For the legacy calibrated analyses, I applied calibrations for three nodes in Eleotridae: Butidae (47 Ma), Eleotridae (46 Ma), and the clade containing (Odontobutidae, Milyeringidae, Butidae, Eleotridae: 65 Ma). These calibrations were derived from the larger-scale analysis of Gobiiformes in [23], and I applied them as normal priors, with standard deviations of 10 Ma, more than encompassing the 95% highest posterior densities of the calibration estimates in the original analysis. For Apogonidae, I dated the root of the family based on the calibrated gobiiform phylogeny of [23], at 51 Ma, also applied as a normal prior with a conservative 10 Ma standard deviation. The Bayesian search ran for 10.0 × 10^8^ generations (Eleotridae) or 10.0 × 10^9^ generations (Apogonidae), with trees sampled every 1000 or 10,000 generations, respectively, and resulting in a pool of 100,000 trees. At the end of the analyses, estimated effective sample sizes (ESS) for all parameters exceeded 200 for Eleotridae, and all save the overall posterior and prior for Apogonidae (ESS of 171 and 169, respectively), likely due to weakly supported resolution along part of the phylogenetic backbone. I confirmed that 10% was the appropriate burn-in fraction with Tracer 1.5, then constructed a maximum clade credibility consensus of the post burn-in trees using TreeAnnotator 1.7.5 [16], and visualized this tree using FigTree 1.3.1 [24]. For the isthmus calibrated analyses, I assigned a lognormal prior for the youngest transisthmian split in Eleotridae, between *Dormitator* species, and for the origin of the Eastern Pacific clade in Apogonidae. For each calibration, I specified an offset of 3.1 (representing the hard minimum of 3.1 Ma for transisthmian divergences), and standard deviation of 1.0. I ran and sampled these analyses as for the legacy calibrated hypotheses, and in both cases, ESS values well exceeded 200. I also constructed and visualized the consensus hypothesis as described above for the legacy calibrated hypotheses.

### Morphometric data and analyses

**Fig. 1:**
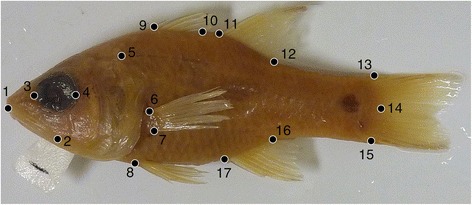
Locations of morphometric landmarks, shown on left lateral view of *Apogon imberbis* (UF 225062). Landmarks digitized are: (1) anterior tip of premaxilla, (2) posteroventral tip of maxilla, (3) anterior edge of eye, (4) posterior edge of eye, (5) anterior extent of opercular opening, (6) dorsal extent of pectoral fin base, (7) ventral extent of pectoral fin base, (8) anterior extent of pelvic fins, (9) base of anteriormost dorsal spine in first dorsal fin, (10) base of posteriormost dorsal spine in first dorsal fin, (11) base of anteriormost dorsal spine in second dorsal fin, (12) base of posteriormost dorsal ray of second dorsal fin, (13) dorsal tip of caudal fin hypurals, (14) center of caudal fin hypurals, (15) vental tip of caudal fin hypurals, (16) base of posteriormost anal fin ray, (17) base of first anal spine

To quantify phenotypic variation between geminate species, I examined preserved specimens of Eleotridae in each of the six species comprising the three geminate pairs in *Eleotris*, *Dormitator*, and *Gobiomorus*. *Leptophilypnus*, the other geminate pair present in the phylogenetic analysis, was not used for shape comparisons because adults of *Leptophilypnus* are too small (maximum 64 mm SL, most much smaller [25]) to be reliably photographed and landmarked. For Apogonidae, I examined representatives of the entire New World clade, including Caribbean species, the Mediterranean singleton *Apogon imberbis*, and the clade of Eastern Pacific taxa. Species and specimens examined are listed in Additional file 1: Table S3; individuals were initially fixed in formalin and preserved in 70% ethanol. I examined 75 individuals of Eleotridae (range of six to 16 individuals for each of the six species) and 157 individuals of Apogonidae (range of two to 11 individuals for each of 25 species, including several not present in the molecular phylogeny). In every case, I selected unbent, intact adults. For each individual, I photographed the specimen using a Panasonic Lumix DMC-ZS3 digital camera mounted on a copystand. I then digitized a suite of 17 landmarks from the left side of each individual (Fig. 1), using ImageJ version 1.49 t [26]. These landmarks primarily describe the shape of the body, including the placements of the median fins, but also include the dimensions of the mouth and eyes, the anterior extent of the opercular opening, and the positions of the pelvic and pectoral fins. All landmarks were assigned in all specimens.

To determine whether or not Caribbean and Eastern Pacific taxa among Apogonidae and Eleotridae are separated in shape space, I used MorphoJ version 1.05d [27]. I forwarded landmark coordinates to MorphoJ, where I performed a Procrustes fit, generated a covariance matrix, and used that matrix as data for principal components analysis (PCA) and canonical variates analysis (CVA). For the CVA, I grouped the individuals by geographic range. I also used MorphoJ to generate plots of the PCA for both sleepers and cardinalfishes. To evaluate whether or not the shape differences among transisthmian taxa are significant, I performed Procrustes PCA and MANOVA on the morphometric landmark data with range as a factor, using the R (version 3.2.1) package *geomorph* (version 2.1.5 [28]).

### Phylogenetic comparative analyses

The transisthmian divergences among Eleotridae are all phylogenetically independent. In contrast, among New World Apogonidae the Eastern Pacific radiation is nested inside a Caribbean clade, along with a single Mediterranean species. With this pattern, comparisons among species inhabiting different ranges are not independent, and so I used phylogenetic PCA and MANOVA to correct for shared evolutionary history. For these analyses, I used a reduced subset of the cardinalfish morphometric data that only included species present in the phylogenetic tree (17 species overlapped between the morphometric and phylogenetic analyses). Using that phylogeny, I first tested for phylogenetic signal in the cardinalfish morphometric data with *geomorph*. I then performed a phylogenetic MANOVA two ways. First, using the uncorrected PC scores for the reduced taxon set, I executed a phylogenetic MANOVA of shape change by range with the *geiger* package (version 2.0.6 [29]), and assessed significance of range as a factor using the Wilks test. Then, I generated a phylogenetic PCA of the shape coordinates for the reduced taxon set with the *phytools* package (version 0.5–20 [30]). Finally, I performed a phylogenetic MANOVA of the phylogenetically corrected PC scores, again using *geiger*.

To gauge whether or not the rate or mode of phenotypic change in Apogonidae shifted across the phylogeny when lineages were sundered by closure of the Isthmus of Panama, I used the R package *mvMORPH* [31]. I reconstructed the evolution of geographic range (Caribbean, Eastern Pacific, or Mediterranean) on the calibrated phylogeny, as well as scores for the first three PC axes, and then fitted multivariate models of trait evolution to the reconstructions. I first calculated the fit of single and multiple rate Brownian Motion (BM; trait variance increases over time, without constraint) and Ornstein-Uhlbeck (OU; trait variance is constrained about a mean, emulating selection) models across the phylogeny. Then, I imposed models incorporating mode shifts at the closure of the Isthmus of Panama (separation of the Eastern Pacific clade from the Caribbean radiation). I applied six mode shift models, the first four incorporating shifts between BM and OU modes: ecological release (shift from OU -> BM process with same rate); release and radiate (shift from OU -> BM process with variable rates); ecological constraint (shift from BM -> OU process with same rate); and radiation and ecological constraint (shift from BM -> OU process with variable rates [32]). These models emulate scenarios in which character evolution becomes more (ecological constraint) or less (ecological release) constrained following the splitting of populations by the closure of the Isthmus of Panama and associated environmental changes.

I also tested two models involving mode switches between BM and EB (early burst, a modification of the accelerating/decelerating rates [ACDC] model of character change in which the rate of evolution slows exponentially, emulating an adaptive radiation [33, 34]). These models are denoted Brownian motion/early burst (BMEB) and early burst/Brownian motion (EBBM) in [31], and in this context would describe adaptive radiations taking place before or after the populations were split by the Isthmus of Panama. Finally, I evaluated AIC and AIC_c_ score values for each of the ten evolutionary models to assess the fits, and compared support among models using Akaike weights computed from the AIC_c_ scores [35]. I performed each of these analyses on both the legacy calibrated and isthmus calibrated phylogenies, to compare the effects of different calibration regimes.

## Results

### Phylogeny and transisthmian divergences

**Fig. 2:**
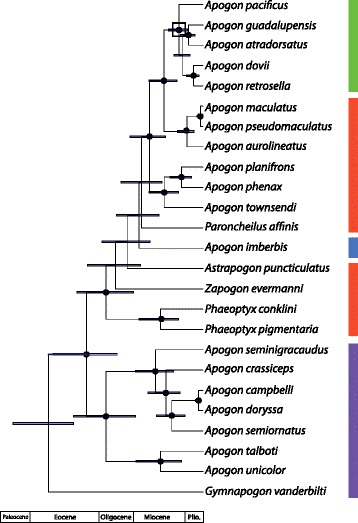
Phylogeny of Apogonidae, based on fragments of one mitochondrial (COI) and two nuclear (ENC1 and RAG1) genes and calibrated with fossil-based legacy dates. *Error bars* indicate 95% highest posterior density, and *black circles* at nodes indicate 95–100% posterior probability. For compactness, the most distal outgroup, *Pseudamia gelatinosa*, is not shown. *Colored bars* indicate ranges: Caribbean = *red*, Eastern Pacific = *green*, Mediterranean = *blue*, Indo-Pacific = *purple*. *Black box* at node indicates separation of lineages by the Isthmus of Panama

**Fig. 3:**
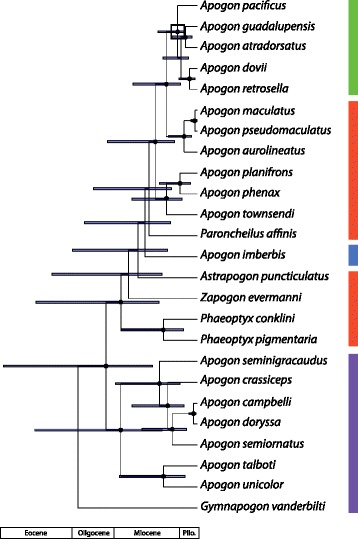
Phylogeny of Apogonidae, based on fragments of one mitochondrial (COI) and two nuclear (ENC1 and RAG1) genes and calibrated with minimum date of closure of the Isthmus of Panama. *Error bars* indicate 95% highest posterior density, and *black circles* at nodes indicate 95–100% posterior probability. For compactness, the most distal outgroup, *Pseudamia gelatinosa*, is not shown. *Colored bars* indicate ranges: Caribbean = *red*, Eastern Pacific = *green*, Mediterranean = *blue*, Indo-Pacific = *purple*. *Black box* at node indicates separation of lineages by the Isthmus of Panama

**Fig. 4:**
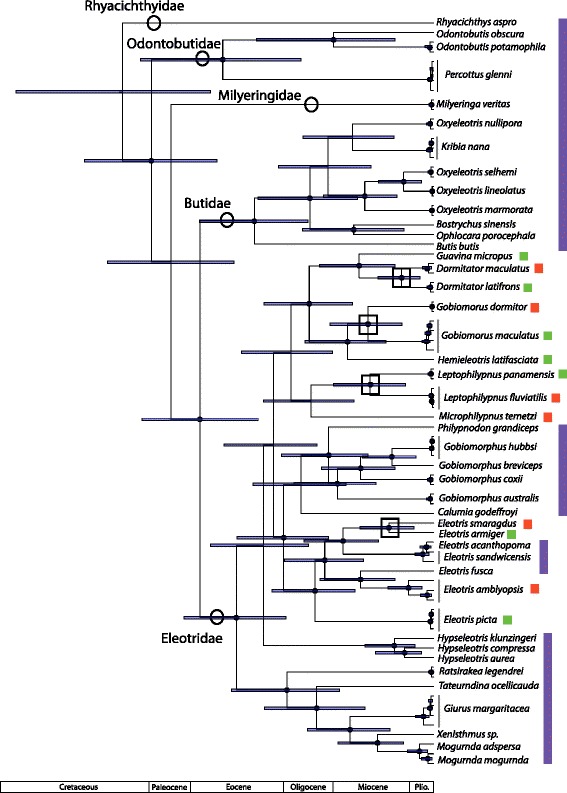
Phylogeny of Eleotridae, based on four mitochondrial (cytb, COI, ND1, ND2) genes and calibrated with fossil-based legacy dates. *Error bars* indicate 95% highest posterior density, and *black circles* at nodes indicate 95–100% posterior probability. Familial classification is indicated on clades, and *colored bars* indicate ranges: Caribbean = *red*, Eastern Pacific = *green*, Indo-Pacific = *purple*. *Black boxes* at nodes indicate separation of lineages by the Isthmus of Panama. In the *Eleotris* clade, other geminate splits than the one examined here have occurred, but were not included in this analysis

**Fig. 5:**
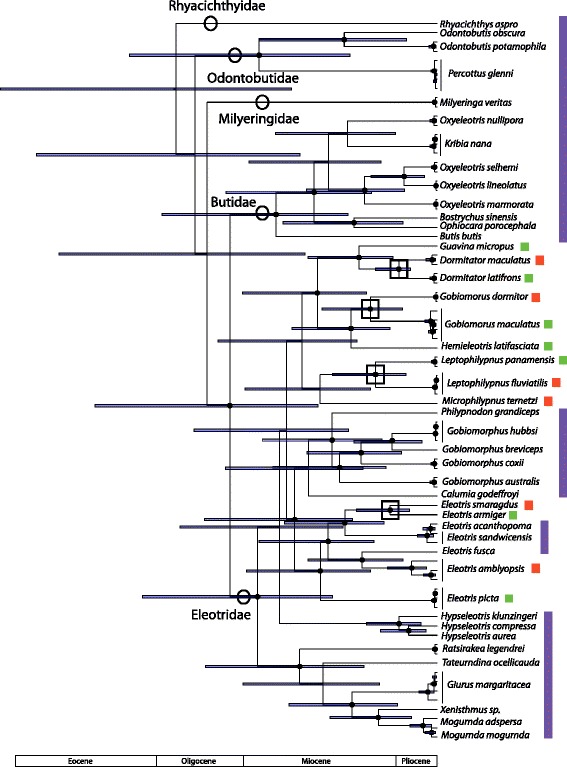
Phylogeny of Eleotridae, based on four mitochondrial (cytb, COI, ND1, ND2) genes and calibrated with minimum date of closure of the Isthmus of Panama. *Error bars* indicate 95% highest posterior density, and *black circles* at nodes indicate 95–100% posterior probability. Familial classification is indicated on clades, and *colored bars* indicate ranges: Caribbean = *red*, Eastern Pacific = *green*, Indo-Pacific = *purple*. *Black boxes* at nodes indicate separation of lineages by the Isthmus of Panama. In the *Eleotris* clade, other geminate splits than the one examined here have occurred, but were not included in this analysis

The legacy and isthmus calibrated phylogenies inferred for Apogonidae are shown in Figs. 2 and 3, and for Eleotridae in Figs. 4 and 5; note the differing timescales and the larger error bars in the isthmus calibrated analyses. In both cases, the hypotheses generally agree well with previously published studies. For Apogonidae, the hypothesis agrees in all major respects with the relationships inferred in [10], including the presence of separate clades for Indo-Pacific and New World + Mediterranean *Apogon*, and the nesting of *Astrapogon*, *Phaeoptyx*, *Paroncheilus*, and *Zapogon* within the *Apogon* clade. The five Eastern Pacific *Apogon* species form a clade, and that clade is nested within the Atlantic and Mediterranean species of *Apogon*. The hypotheses differ slightly in the relationships among *Phaeoptyx*, Eastern Pacific *Apogon*, *A. aurolineatus*, and the Mediterranean *A. imberbis*, generally where nodal support is the weakest.

Similarly, Eleotridae relationships recovered here are mostly congruent with those of [9, 12, 13], but differ slightly along the backbones, areas of weaker support, particularly as compared to the parsimony-based analysis of [9]. This hypothesis resolves two New World clades, one including *Eleotris*, and a second containing *Dormitator*, *Guavina*, *Hemieleotris*, *Gobiomorus*, *Microphilypnus*, and *Leptophilypnus*. Transisthmian sister taxa of Eleotridae in this hypothesis are present in the genera *Dormitator*, *Eleotris*, *Gobiomorus*, and *Leptophilypnus*.

In both Apogonidae and Eleotridae, legacy calibrated phylogenies indicate that the New World lineages arose in the Oligocene, between 27.4–37.2 Ma, and that transisthmian splits were established across a range of divergences spanning 7.4–15.1 million years in the middle to late Miocene. The isthmus calibrated phylogenies indicate shallower divergence dates overall, with New World lineages postulated to have arisen from the latest Oligocene into the Miocene, between 16.2–24.2 Ma, and transisthmian splits occurring from 5.1 to 9.9 Ma (late Miocene to early Pliocene; Table 1). Isthmus calibrated nodes have greater uncertainty than those in the legacy calibrated hypothesis, because the single, relatively young calibration estimate yields greater error deeper in the phylogeny, more distant from the calibrated node and in areas of weaker support. Multiple deeper legacy calibrations allow more precise dating estimates. The complementary median divergence times inferred in both phylogenies are within each other’s error estimates, particularly among the more recent splits.

**Table 1:** Inferred ages in Ma and 95% highest posterior density (in parentheses) for clades in Eleotridae and Apogonidae, determined from both legacy and isthmus calibrations. *Eleotris* and *Dormitator*/*Gobiomorus* clade ages are for the larger New World clades containing those genera

Node	Age - legacy	Age - isthmus
Eleotridae: *Dormitator maculatus/latifrons*	7.4 (3.4–12.8)	5.1 (3.2–10.6)
Eleotridae: *Eleotris smaragdus/armiger*	10.3 (5.3–17.0)	6.4 (2.0–15.7)
Eleotridae: *Gobiomorus dormitor/maculatus*	15.1 (7.7–24.5)	9.9 (3.2–23.8)
Eleotridae: *Leptophilypnus fluviatilis/panamensis*	14.6 (6.8–23.6)	9.2 (2.7–22.3)
Apogonidae Eastern Pacific clade stem	12.5 (8.2–17.3)	8.2 (4.4–17.0)
Apogonidae Eastern Pacific clade crown	7.6 (4.9–10.9)	5.1 (3.1–10.5)
New World Eleotridae: *Eleotris* clade stem	34.5 (24.2–45.4)	20.2 (7.6–46.7)
New World Eleotridae: *Eleotris* clade crown	27.4 (18.1–37.2)	16.3 (5.6–37.7)
New World Eleotridae: *Dormitator*/*Gobiomorus* clade stem	32.9 (23.3–44.4)	19.3 (7.4–44.7)
New World Eleotridae: *Dormitator*/*Gobiomorus* clade crown	28.7 (18.7–39.1)	17.2 (6.8–39.8)
New World Apogonidae clade stem	37.2 (27.4–47.9)	24.2 (11.9–51.4)
New World Apogonidae clade crown	31.0 (22.2–40.3)	20.28 (10.12–42.92)

### Morphometric PCA, CVA, and MANOVA

**Fig. 6:**
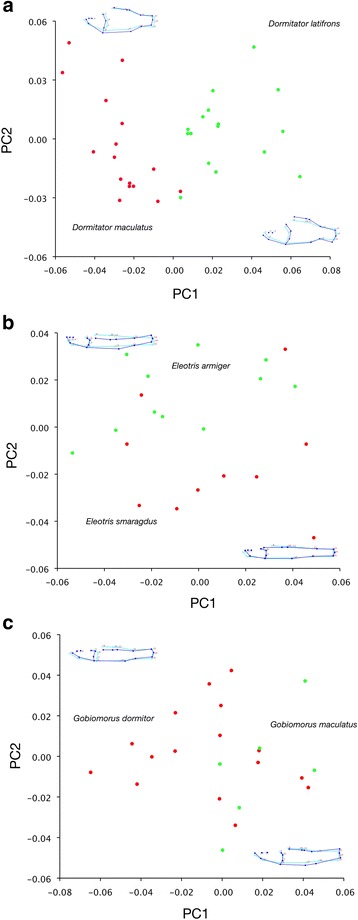
Graphs of PC1 vs. PC2 for geminate species of Eleotridae. **a** *Dormitator*, **b** *Eleotris*, and **c** *Gobiomorus*. Wireframe graphs depict shape change on each axis; *light blue* is the average specimen shape, and *dark blue* is a change of 0.1 unit on that axis. *Green* indicates species is known from the Eastern Pacific, *red* indicates distribution in the Caribbean

Results of the morphometric PCAs for Eleotridae are shown in Fig. 6. In *Dormitator* (Fig. 6a), PC1 and PC2 account for 32% and 16% of the total variance, and both describe changes in the depth of the head and body as well as the width of the head. *Eleotris* PC1 and PC2 are 31% and 20% of the total variance, with PC1 encompassing the relative elongation of the head, and PC2 describing the relative orientations of the head and caudal peduncle (Fig. 6b). For *Gobiomorus*, PC1 and PC2 account for 35% and 20% of the variance, PC1 accounts for relative displacement of the head and caudal peduncle, and PC2 describes changes in body depth and head shape (Fig. 6c). In all three geminate pair comparisons, shape differences between the species are slight, and eigenvalues for each PC are low (maximally 0.001). CVA for geminate pairs of Eleotridae resulted in each case with a single CV axis accounting for 100% of the variation, with wide separation among species in every comparison.

**Fig. 7:**
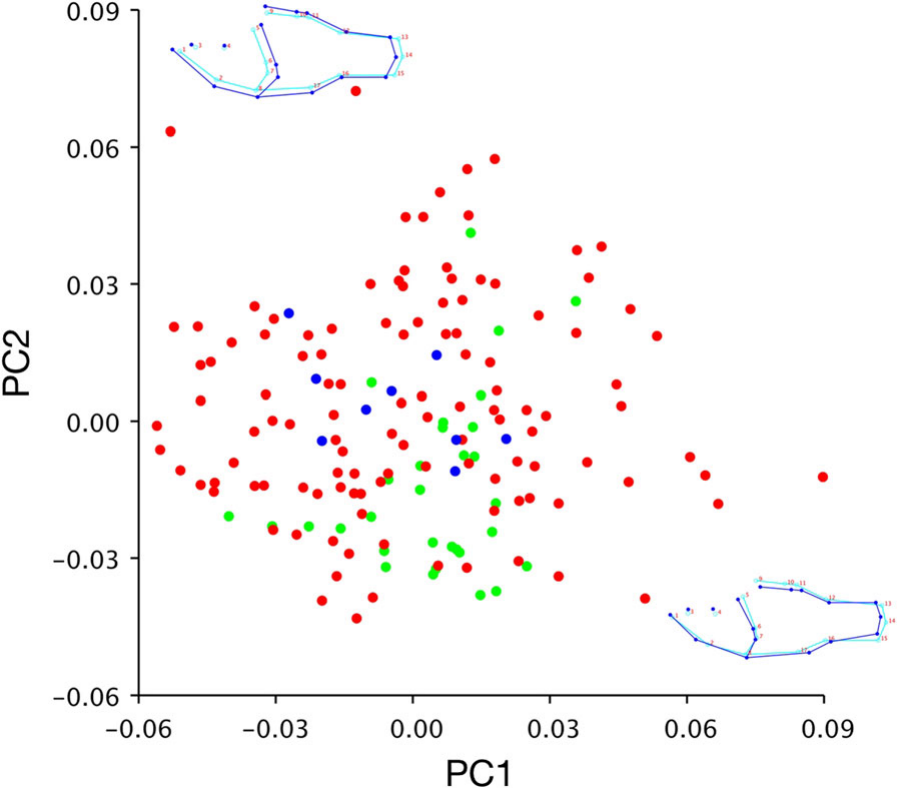
Graphs of PC1 vs. PC2 for New World and Mediterranean Apogonidae. Wireframe graphs depict shape change on each axis; *light blue* is the average specimen shape, and *dark blue* is a change of 0.1 unit on that axis. *Green* indicates species is known from the Eastern Pacific, *red* indicates distribution in the Caribbean, and *blue* indicates the Mediterranean

**Fig. 8:**
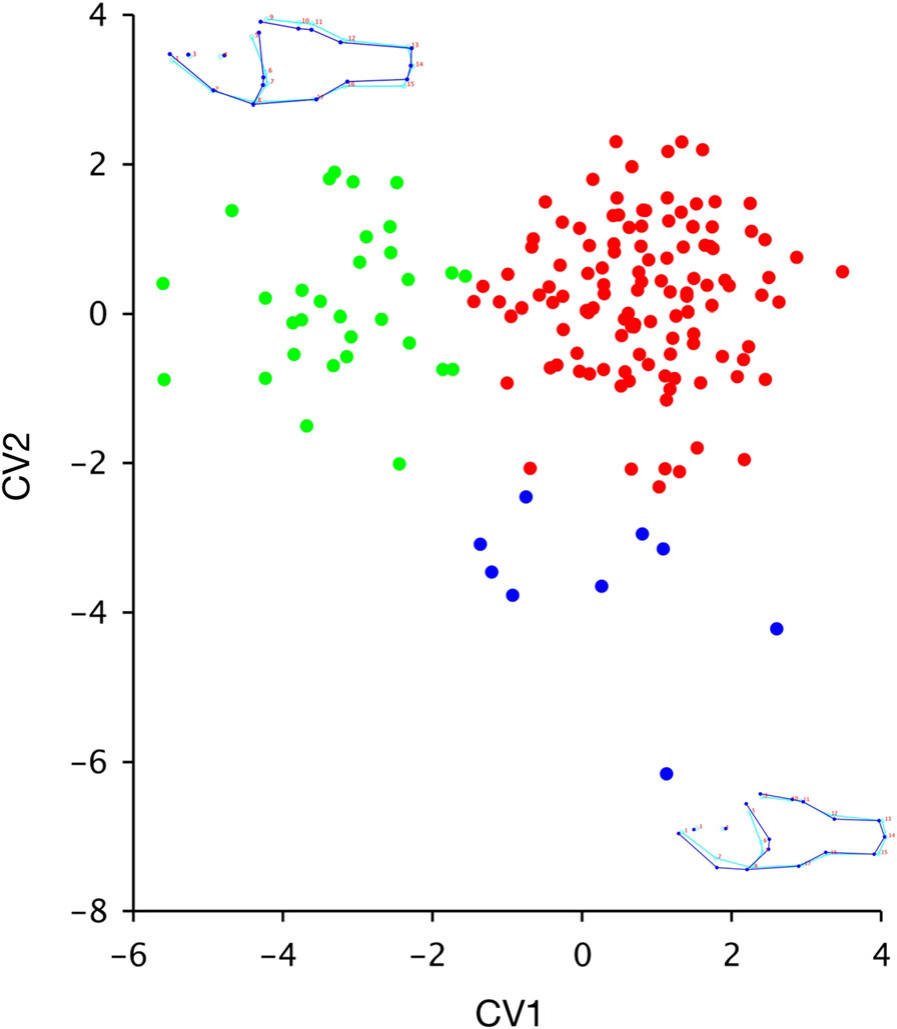
Graphs of CV1 vs. CV2 for New World and Mediterranean Apogonidae. Wireframe graphs depict shape change on each axis; *light blue* is the average specimen shape, and *dark blue* is a change of 0.1 unit on that axis. *Green* indicates species is known from the Eastern Pacific, *red* indicates distribution in the Caribbean, and *blue* indicates the Mediterranean

Morphometric PCA results for Apogonidae are presented in Fig. 7. Among cardinalfishes, the overlap among regions is more complete, with the more numerous Caribbean forms completely containing the variation among those in the Eastern Pacific and Mediterranean. The PC axes also describe more complex variation; PC1 (22% of variation) encompasses a shift in body proportions with a deeper cranial portion, and a slight rotation of the caudal region. PC2 (17% of variation) is more easily interpretable as an enlargement of the head, and deepening and shortening of the posterior portion of the body. The CVA, in which the axes are oriented in order to maximize the differences among regions, shows more separation among the apogonid ranges (Fig. 8). Caribbean and Eastern Pacific species are separated on the CV1 axis, which primarily describes widening and enlargement of the head. All New World taxa are separated from the Mediterranean species on CV2, which describes narrowing of the posterior part of the body, most particularly the caudal peduncle.

Among the eleotrid genera, the first nine PC axes account for 91–94% of the total variance. MANOVA of PC1-PC9 for geminate species by range in *Dormitator* was highly significant (*p* = 0.001), significant in *Eleotris* (*p* = 0.015), and nearly significant for *Gobiomorus* (*p* = 0.07). In Apogonidae, twelve PC axes account for 91% of the variance. MANOVA of these axes among ranges (Caribbean, Eastern Pacific, and Mediterranean) was also highly significant (*p* = 0.001).

I detected phylogenetic signal among the shape data (PC scores) for Apogonidae based on the Kmult test (*p* = 0.031 for both legacy and isthmus calibrated phylogenies). After phylogenetic correction, I found comparisons among ranges to be insignificant based on both a phylogenetic MANOVA of PC scores (first twelve PC axis scores, including 91% of variation and yielding *p* = 0.941 for legacy calibrated phylogeny and *p* = 0.922 for isthmus calibrated phylogeny), and a phylogenetic MANOVA of phylogenetically corrected PC scores (first three PC axis scores, accounting for 93% of the variation, yielding *p* = 0.628 for legacy calibrated phylogeny and *p* = 0.667 for isthmus calibrated phylogeny, both assessed with the Wilks test). Results of MANOVAs are presented in Table 2.

**Table 2:** Results of Procrustes MANOVA of morphometric shape change by range for transisthmian New World Eleotridae and Apogonidae, and phylogenetic MANOVA for both PC scores and phylogenetically corrected PC scores for Apogonidae

Taxon & Analysis	F	P
Procrustes MANOVA		
Eleotridae: *Dormitator*	10.584	**0.001**
Eleotridae: *Eleotris*	2.243	**0.015**
Eleotridae: *Gobiomorphus*	1.645	0.077
Apogonidae	5.472	**0.001**
Phylogenetic MANOVA		
Apogonidae (PC scores, legacy)	1.661	0.941
Apogonidae (PC scores, isthmus)	1.661	0.921
Apogonidae (corrected PC scores, legacy)	1.366	0.628
Apogonidae (corrected PC scores, isthmus)	1.362	0.667

Phylogenetic MANOVAs were calculated for both legacy and isthmus calibrated phylogenies. Significant *P*-values are shown in boldface

### Analyses of phylogenetic rate and mode

Of the models tested, the multiple rate Ornstein-Uhlbeck model was fitted with the highest log-likelihood, lowest AIC and AIC_c_ scores, and by far the highest AIC weight. This model is consistent with selection on cardinalfish phenotypes throughout the period of Isthmus uplift, but with different rates before and after Isthmus closure. Log-likelihoods and AIC scores and weights for each of the legacy and isthmus calibrated phylogenies under the various models are given in Table 3. The relative rates of trait evolution for the Eastern Pacific clade vs. the Caribbean radiation in the multirate Brownian motion model are similar, although differently localized. When calculated with the legacy calibrated phylogeny, rates of change for PC1 are nearly equivalent in the Caribbean and Eastern Pacific, while those of PC2 and PC3 are elevated in the Eastern Pacific. The mean rate of change across the three PC axes is also higher in the Eastern Pacific relative to the Caribbean. Results from calculation with the isthmus calibrated phylogeny are similar, with comparable rates indicated for PC1 in the Caribbean and Eastern Pacific, an elevated rate on PC3 in the Eastern Pacific as compared to the Caribbean, but a higher rate of change on PC2 in the Caribbean. The mean rate of change across the three PC axes is higher overall in the Caribbean under this calibration (Table 4).

**Table 3:** Results of mvMORPH model fitting for transisthmian Apogonidae, showing log-likelihood (LnL), Akaike information criterion (AIC), AIC corrected for limited sampling (AIC_c_), difference between AIC_c_ and AIC_c_ of best fit model (ΔAIC_c_), and AIC weight

Model	LnL	AIC	AIC_c_	ΔAIC_c_	AIC_c_Wt
Legacy calibrated phylogeny					
Brownian motion single rate	123.77	−229.54	−203.82	221	0.00
Brownian motion multiple rate	130.51	−219.03	−403.83	20.99	0.00
Ornstein-Uhlbeck single rate	134.9	−239.91	240.09	664.91	0.00
Ornstein-Uhlbeck multiple rate	**141.01**	**−240.02**	**−424.82**	**0.00**	**0.99**
Ecological release	125.02	−220.04	259.96	684.78	0.00
Release and radiate	135.67	−229.34	−414.14	10.68	0.00
Ecological constraint	119.64	−209.28	270.72	695.54	0.00
Radiation & ecological constraint	126.43	−210.86	−395.66	30.16	0.00
Brownian motion/early burst	122.50	−225.01	−188.34	236.48	0.00
Early burst/Brownian motion	53.24	−86.49	−49.83	374.99	0.00
Isthmus calibrated phylogeny					
Brownian motion single rate	123.94	−229.89	−204.17	223.12	0.00
Brownian motion multiple rate	131.21	−220.42	−405.22	22.07	0.00
Ornstein-Uhlbeck single rate	135.1	−240.19	239.81	667.10	0.00
Ornstein-Uhlbeck multiple rate	**142.24**	**−242.49**	**−427.29**	**0.00**	**0.99**
Ecological release	129.60	−229.20	250.80	678.09	0.00
Release and radiate	135.48	−228.97	−413.77	13.52	0.00
Ecological constraint	123.47	−216.94	263.05	690.34	0.00
Radiation & ecological constraint	125.51	−209.02	−393.82	33.47	0.00
Brownian motion/early burst	121.06	−222.13	−185.47	241.82	0.00
Early burst/Brownian motion	121.78	−223.55	−186.89	240.40	0.00

Most highly favored model is shown in boldface

**Table 4:** Rates of shape change in the first three PC axes for the Caribbean radiation and Eastern Pacific Clade of Apogonidae following separation by the Isthmus of Panama

Area	PC1	PC2	PC3	Mean
Legacy calibrated phylogeny				
Caribbean	3.47E-05	7.21E-06	2.04E-05	2.07E-05
Eastern Pacific	3.72E-05	4.01E-05	3.03E-05	3.59E-05
Isthmus calibrated phylogeny				
Caribbean	5.07E-05	1.07E-04	3.01E-05	6.25E-05
Eastern Pacific	5.16E-05	5.58E-05	4.12E-05	4.97E-05

## Discussion

### Phylogenetic relationships and timing

Phylogenetic patterns among New World Eleotridae and Apogonidae are different, with New World species more phylogenetically dispersed and with more separate, lower diversity instances of transisthmian divergence in Eleotridae as compared to Apogonidae. Seven transisthmian splits are scattered throughout Eleotridae; in Apogonidae, a single New World clade contains multiple species on either side of the Isthmus and includes a Mediterranean species. The species diversity in the New World for both groups is similar: 28 species in Eleotridae compared to 24 in Apogonidae. In Eleotridae, just one or a few species is found on either side of the Isthmus in each geminate split, but in Apogonidae, a single clade contains a broad Caribbean radiation, including a clade of Eastern Pacific species as well as a single Mediterranean species. A review of phylogenetic patterns among Atlantic fish taxa concluded that the apogonid pattern, with diversification taking place well after Isthmus closure rather than exclusively driven by it, is more common among reef fishes [2]. The resolution of a Mediterranean species among New World Apogonidae is consistent with transatlantic dispersal from west to east, as has been inferred for several lineages of reef fishes, including some gobies (close relatives of sleepers and cardinalfishes, [2, 36, 37]).

New World lineages of both Apogonidae and Eleotridae arose at roughly the same time, estimated from the legacy calibrated phylogeny at around 27.4–37.2 Ma in the Oligocene, and from the isthmus calibrated phylogeny at 16.3–24.2 Ma in the late Oligocene to Miocene (Table 1). Over most of this time, North and South America were well-separated, and Asia and Africa were as well, resulting in a continuous band of tropical ocean extending around the globe. Among Gobiiformes, several other instances of New World invasion from the east have occurred in both Gobiidae and Gobionellidae. In some cases (*Stenogobius* lineage of Gobionellidae, *Priolepis* and *Glossogobius* lineages of Gobiidae), species generally have wide ranges, and some include the Western Atlantic and/or Eastern Pacific. Among other lineages (*Lophogobius*, *Gunnellichthys*, and the entire Gobiosomatini, all in Gobiidae), larger New World radiations are present. New World invasions in Gobiidae and Gobionellidae have occurred over a wide span from the mid-Eocene (Gobiosomatini) through to the mid-Miocene, when the Terminal Tethyan Event (collision of Africa and Asia) closed off passage between the Indian Ocean and the Atlantic [38].

Splits among geminate lineages in Apogonidae and Eleotridae vary in their timing, but all occurred in the late Miocene to early Pliocene, estimated from the legacy calibrated phylogeny between 7.4 and 15.1 Ma, and from the isthmus calibrated phylogeny at 5.1–9.9 Ma (Table 1). These dates all predate the canonical date of final closure of the Isthmus of Panama at 3.1 Ma, but correspond roughly to one of the two dates inferred by [4] for marine vicariance associated with Isthmus uplift. In that study, a review of both marine and terrestrial data indicated that the Isthmus of Panama was established enough, even if it was not completely closed, to split marine populations at two times in the Miocene, first at 23 Ma and then again at 7 Ma. Extinctions among transisthmian taxa following Isthmus closure may also affect the inference of split dates, with earlier divergences appearing to be geminates if closer relatives have since become extinct.

Among both Eleotridae and Apogonidae, the legacy calibrated estimates across the tree have lower error estimates (95% highest posterior density) and are in much better agreement with previous phylogenies of gobiiformes [23, 28] as well as larger phylogenies of fishes [39–41]. The lower error estimates in the legacy calibrated phylogeny are at least partially due to application of the calibrations at deeper nodes in the phylogeny, and in the case of Eleotridae, to the use of multiple calibrations. The legacy calibrated estimates are probably the more accurate inferences. Errors propagated by legacy calibrations are small [9], and are generally preferable to using a biogeographic calibration, particularly when the legacy estimates derive from well-dated fossils and are applied at multiple nodes. Also, fossils provide truly independent data for calibration estimates. A calibration based on a biogeographic event is independent from organismal phylogeny, however given that organismal evolutionary patterns may be greatly affected by biogeographic events, and also that phylogenies are often themselves used to corroborate the dating of such events, the use of fossil based calibrations, even legacy calibrations, is preferable.

Ecological tolerance among the transisthmian lineages does not correlate with divergence time. New World species of Apogonidae (genera *Apogon*, *Astrapogon*, *Phaeoptyx*, *Paroncheilus,* and *Zapogon*) are all marine, found in shallow coral or rocky reef habitats in their respective seas [42, 43]. Eleotridae are generally more euryhaline, and inhabit brackish and freshwater as well as salt. *Eleotris* are found in nearshore fresh and brackish habitats, including mangrove stands, as well as shallow marine water. *Dormitator* inhabit marshes, estuaries, and rivers, and can tolerate seawater. *Gobiomorus* are primarily found in freshwater rivers and lakes, including landlocked populations, but may also inhabit brackish habitats [44]. With the Isthmus of Panama gradually uplifting, likely in several separate blocks, it follows that the deeper-dwelling marine species would be separated first, followed by shallower ones, and then finally the fresh to brackish water species would diverge as their drainage systems separated into westward and eastward flowing, a pattern detected among multiple pairs of transisthmian snapping shrimp [45]. I did not detect that pattern among sleepers or cardinalfishes. The wholly marine Apogonidae show divergence of the Eastern Pacific clade from their Caribbean relatives at 12.5 Ma (legacy dating)/8.2 Ma (isthmus dating), with isolation of the crown Eastern Pacific clade complete by 7.6 Ma (legacy dating)/5.1 Ma (isthmus dating). *Eleotris*, the genus with the highest saltwater tolerance among those examined, includes a geminate divergence date estimate of 10.3 Ma (legacy)/ 6.4 (isthmus). Intermediate is *Dormitator*, with the youngest estimated divergence of 7.6 Ma (legacy)/ 5.1 (isthmus), and the most freshwater pair, in *Gobiomorus*, exhibits the oldest estimated split date at 15.1 Ma (legacy)/ 9.9 (isthmus). There is no clear pattern among these taxa relating salt tolerance to transisthmian divergence, but given that the closure of the Isthmus was a complex, protracted process, it is not surprising that the responses of animal taxa affected would be idiosyncratic and variable.

### Patterns and rates of transisthmian morphological divergence

Geometric morphometric data provide an overall assessment of comparative shape change, and are useful for revealing subtle shifts in organismal morphology. Among the transisthmian lineages examined here, expressions of morphological change are mosaic and varied, and do not show a common pattern among disparate taxa. In Eleotridae, transisthmian pairs show changes in head depth and width as well as body depth in *Dormitator*; head elongation and relative head/tail proportions in *Eleotris*; and a combination of overall width and head/tail displacement in *Gobiomorus*. All the changes are slight, and do not seem to reflect a single overarching pattern of change among all the pairs. The changes are statistically significant by range in the MANOVA (or nearly so in *Gobiomorus*), but not consistent among geminates. I did not apply phylogenetic correction to the independent transisthmian comparisons in Eleotridae, but for the nested patterns in Apogonidae, I applied phylogenetic corrections both to the PCA and to the MANOVA, using a subset of Apogonidae for which I had both molecular and morphometric data. After correction for the nested phylogenetic relationships, MANOVAs of comparisons among ranges were not significant.

The few previous studies of morphological change in transisthmian taxa have yielded similar results, with geminate species generally being morphometrically distinguishable, but without clear trends in shape or size change [6–8, 46]. Rather than phenotypic differences, the primary consistent distinction among the transisthmian marine taxa reviewed by [3], was a change in maternal provisioning of eggs, such that eggs in the higher primary productivity environment (Eastern Pacific) were smaller and had lower maternal provisioning than those in the more nutrient-poor regime (Caribbean). This trend appears to hold not just for modern taxa, but for fossil species as well, with egg size decreasing in the Eastern Pacific roughly two million years ago [47]. In the current analysis, I found that the best-fitting model of morphological change, regardless of phylogenetic calibration method, postulated a rate shift at closure of the Isthmus of Panama, and modeled change as variation around a selective optimum (Ornstein-Uhlbeck).

A final, potentially very important factor shaping patterns of morphology and ecology across the Isthmus is extinction. Ecologically, generalist taxa are less susceptible to extinction than specialists when confronted with habitat change [48]. Sleepers and cardinalfishes are both widely distributed groups, with high diversification rates (Apogonidae, [23]), wide ecological tolerances (Eleotridae, [44]), and generalist feeding strategies [42–44]. Although impossible to measure from phylogenetic reconstructions based on extant taxa, extinction of more specialized or canalized phenotypes would be expected in a period of tectonic upheaval and environmental change. In the cases of Apogonidae and Eleotridae, their generalist ecological tolerances likely contributed to their persistence and radiation on both sides of the Isthmus of Panama, even after Isthmus closure and associated environmental change.

## Conclusions

Calibrated organismal phylogenies have been used extensively to estimate when marine populations were split by the closure of the Isthmus of Panama, with results indicating most commonly that populations diverged earlier than the canonical data of final Isthmus closure at 3.1 Ma [3]. Ongoing geological and evolutionary studies are revealing that the closure of the Isthmus was a complex tectonic event, with varied effects on marine (and terrestrial, [4]) faunas. I show that for two families of fishes, Eleotridae and Apogonidae, transisthmian lineages split at different times throughout the Miocene, that the divergence ages are not correlated to ecology, and that morphometric distinctions are detectable among geminate taxa. I estimate the timing of phylogenetic events using both legacy fossil based calibrations and a biogeographic calibration, and find that the legacy calibrations produce more precise dates that are more congruent with estimates from previous studies. I also demonstrate that among Apogonidae, fits of evolutionary models to the phylogeny show that rates of morphometric change shifted at Isthmus closure, variably among component axes of change, with acceleration of the rate of shape change among taxa in the Eastern Pacific.

## Additional file

Additional file 1: Table S1. Sequences used for phylogenetic analysis of Eleotridae; GenBank accession numbers are listed for each gene. **Table S2.** Sequences used for phylogenetic analysis of Apogonidae; GenBank accession numbers are listed for each gene. **Table S3.** Species examined for morphometric analysis. Car = Caribbean; EPac = Eastern Pacific; MED = Mediterranean; UF = Florida Museum of Natural History. (DOCX 115 kb)

## Abbreviations

ANSP: Academy of Natural Sciences, Philadelphia, Pennsylvania, USA
FMNH: Field Museum of Natural History, Chicago, Illinois, USA
LACM: Natural History Museum of Los Angeles County, Los Angeles, California, USA
UF: Florida Museum of Natural History, Gainesville, Florida, USA
