# Supplementary material for: Patterns of divergence in fish species separated by the Isthmus of Panama
Source: BMC Evol Biol. 2017 May 10;17:111. doi: 10.1186/s12862-017-0957-4 (PMC5424344; doi:10.1186/s12862-017-0957-4)
Supplement: Additional file 1: Table S1. — Sequences used for phylogenetic analysis of Eleotridae; GenBank accession numbers are listed for each gene. Table S2. Sequences used for phylogenetic analysis of Apogonidae; GenBank accession numbers are listed for each gene. Table S3. Species examined for morphometric analysis. Car = Caribbean; EPac = Eastern Pacific; MED = Mediterranean; UF = Florida Museum of Natural History. (DOCX 115 kb) [file 12862_2017_957_MOESM1_ESM.docx]

Table S1: GenBank numbers for Eleotridae used in phylogenetic analysis.

Taxon COI cytb ND1 ND2

Rhyacichthyidae

*Rhyacichthys aspro* AP004454 AP004454 AP004454 AP004454

Odontobutidae

*Odontobutis obscura* AF391330 ------- AF391402 AF391474

*Odontobutis potamophila* AY722174 AY722247 AY722311 AY722371

AY722153 AY722225 AY722290 AY722353

Milyeringidae

*Milyeringa veritas* AY722168 AY722240 AY722305 -------

AY722169 AY722241 AY722306 -------

Butidae

*Bostrychus sinensis* AY722164 AY722236 AY722301 -------

*Butis butis* AY722180 ------- AY722319 AY722377

*Kribia nana* ------- AY722211 AY722278 -------

AY722150 AY722222 AY722288 -------

------- AY722221 AY722287 -------

*Ophiocara porocephala* AY722250 ------- AY722314 -------

*Oxyeleotris lineolatus* AY722165 AY722237 AY722302 AY722364

AY722139 AY722209 AY722276 AY722340

*Oxyeleotris marmorata* AY722177 AY722252 AY722316 AY722374

AY722176 AY722251 AY722315 AY722373

*Oxyeleotris nullipora* ------- AY722249 AY722313 -------

------- AY722242 AY722307 -------

*Oxyeleotris selhemi* AY722179 ------- AY722318 AY722376

AY722166 AY722238 AY722303 AY722365

Eleotridae

*Calumia godfrayi* AY722125 AY722194 AY722262 AY722325

*Dormitator latifrons* AY722142 AY722213 AY722280 AY722343

AY722138 AY722207 AY722274 AY722338

*Dormitator maculatus* AY722143 AY722214 AY722281 AY722344

AY722137 AY722206 AY722273 AY722337

*Eleotris acanthopoma* AP004455 AP004455 AP004455 AP004455

*Eleotris armiger* AY722167 AY722239 AY722304 AY722366

*Eleotris amblyopsis* AY722136 AY722205 AY722272 AY722336

AY722154 AY722226 AY722291 AY722354

AY722141 AY722212 AY722279 AY722342

*Eleotris fusca* AY722172 AY722245 AY722309 AY722369

*Eleotris picta* AY722148 AY722219 AY722286 AY722349

AY722135 AY722204 AY722271 AY722334

AY722157 AY722229 AY722294 AY722357

*Eleotris sandwicensis* AF391477 AY722186 AF391333 AF391405

AF391478 ------- AF391334 AF391406

*Eleotris smaragdus* AF391499 AY722185 AF391355 AF391427

*Gobiomorphus australis* AY722145 AY722216 AY722283 AY722346

AY722147 AY722218 AY722285 AY722348

*Gobiomorphus breviceps* AY722152 AY722224 AY722289 AY722352

*Gobiomorphus coxii* AY722149 AY722220 ------- AY722350

AY722151 AY722223 ------- AY722351

*Gobiomorphus hubbsi* AY722155 AY722227 AY722292 AY722355

AY722156 AY722228 AY722293 AY722356

AY722158 AY722230 AY722295 AY722358

*Gobiomorus dormitor* AY722144 AY722215 AY722282 AY722345

AY722134 AY722203 AY722270 AY722334

*Gobiomorus maculatus* AY722132 AY722201 AY722269 AY722332

AY722124 AY722193 AY722261 AY722324

AY722178 ------- AY722317 AY722375

AY722133 AY722202 -------- AY722333

*Guavina micropus* AY722131 AY722200 AY722268 AY722331

*Hemieleotris latifasciatus* AY722173 AY722246 AY722310 AY722370

*Hypseleotris aurea* AF391536 AY722187 AF391392 AF391464

*Hypseleotris compressa* AF391510 AY722188 AF391366 AF391438

*Hypseleotris klunzingeri* AF391537 AY722189 AF391393 AF391465

*Leptophilypnus fluviatilis* AY722130 AY722199 AY722267 AY722330

AY722129 AY722198 AY722266 AY722329

AY722128 AY722197 AY722265 AY722328

*Leptophilypnus panamensis* AY722127 AY722195 AY722264 AY722326

AY722126 AY722196 AY722263 AY722327

*Microphilypnus ternetzi* AY722181 AY722253 AY722320 AY722378

*Mogurnda adspersa* AF391511 AY722184 AF391367 AF391439

*Mogurnda mogurnda* AY722140 AY722210 AY722277 AY722341

AY722123 AY722192 AY722260 AY722323

*Ophieleotris aporos* AF391512 ------- AF391368 AF391440

AY722160 AY722232 AY722297 AY722360

AY722159 AY722231 AY722296 AY722359

AY722161 AY722233 AY722298 AY722361

*Philypnodon grandiceps* AF391530 ------- AF391386 AF391458

*Ratsirakea legendrei* AY722162 AY722234 AY722299 AY722362

AY722163 AY722235 AY722300 AY722363

*Tateurndina ocellicauda* AY722175 AY722248 AY722312 AY722372

*Xenisthmus* sp. AF391516 ------- AF391372 AF391444

Table S2: GenBank numbers for Apogonidae used in phylogenetic analysis.

Taxon COI ENC1 RAG1

*Apogon atradorsatus* AB890008 AB893478 AB893361

*Apogon aurolineatus* AB890009 ------- AB893362

*Apogon campbelli* AB890010 AB893480 AB893363

*Apogon crassiceps* AB890011 AB893481 AB893364

*Apogon doryssa* AB890012 AB893482 AB893365

*Apogon dovii* ------- AB893483 AB893366

*Apogon guadalupensis* AB890013 AB893484 AB893367

*Apogon imberbis* ------- AB893485 AB893368

*Apogon maculatus* AB890014 AB893486 AB893369

*Apogon pacificus* AB890015 AB893487 AB893370

*Apogon phenax* AB890016 AB893488 AB893371

*Apogon planifrons* AB890017 AB893489 AB893372

*Apogon pseudomaculatus* ------- AB893490 AB893373

*Apogon retrosella* AB890018 AB893491 AB893374

*Apogon seminigracaudus* AB890019 AB893492 AB893375

*Apogon semiornatus* AB890020 AB893493 AB893376

*Apogon talboti* AB890021 AB893494 AB893377

*Apogon townsendi* AB890022 AB893495 AB893378

*Apogon unicolor* AB890023 AB893496 AB893379

*Astrapogon puncticulatus* AB890030 AB893503 AB893386

*Gymnapogon vanderbilti* AB890003 AB893473 AB893356

*Paroncheilus affinis* AB890088 AB893560 AB893440

*Phaeoptyx conklini* AB890089 AB893561 AB893441

*Phaeoptyx pigmentaria* AB890090 AB893562 AB893442

*Pseudamia gelatinosa* AB890005 AB893475 AB893358

*Zapogon evermanni* AB890118 AB893590 AB893468

Table S3: Eleotridae and Apogonidae specimens used in morphometric analyses. ANSP = Academy of Natural Sciences, Philadelphia; CAR = Caribbean/Western Atlantic; EPAC = Eastern Pacific; FMNH = Field Museum of Natural History; LACM = Natural History Museum of Los Angeles County; MED = Mediterranean; UF = Florida Museum of Natural History.

Taxon N Range Catalog number

Eleotridae

*Dormitator latifrons* 1 EPAC LACM 2811

5 EPAC LACM 56197-4

5 EPAC UF 15276

5 EPAC UF 19853

*Dormitator maculatus* 2 CAR LACM 1445

3 CAR LACM 5659

1 CAR LACM 32374-7

10 CAR UF 188083

*Eleotris armiger* 4 EPAC LACM 7037

3 EPAC UF 4465

4 EPAC UF 224455

*Eleotris smaragdus* 5 CAR LACM 31007-21

2 CAR UF 18289

1 CAR UF 62243

1 CAR UF 99915

1 CAR UF 200732

*Gobiomorus dormitor* 3 CAR LACM 32345-4

1 CAR LACM 32347-1

2 CAR LACM 32437-3

1 CAR UF 9263

3 CAR UF 11078

2 CAR UF 11192

4 CAR UF 134876

*Gobiomorus maculatus* 1 EPAC LACM 2776

5 EPAC LACM 4843

Apogonidae

*Apogon atradorsatus* 6 EPAC LACM 43680-14

LACM 45585-3

*Apogon aurolineatus* 6 CAR ANSP 108410

ANSP 1118444

ANSP 127857

*Apogon binotatus* 9 CAR ANSP 94861

ANSP 147262

*Apogon dovii* 7 EPAC LACM 32492-17

LACM 32503-6

LACM 32537-15

*Apogon guadalupensis* 6 EPAC LACM 31782-49

LACM 32097-54

*Apogon imberbis* 9 MED UF 225062

*Apogon lachneri* 5 CAR LACM 4539

LACM 30259-17

*Apogon maculatus* 7 CAR LACM 7742

LACM 36219-4

*Apogon mosavi* 6 CAR ANSP 113434

*Apogon pacificus* 5 EPAC LACM 31776-29

LACM 32495

*Apogon phenax* 7 CAR FMNH 98106

FMNH 98236

FMNH 98409

FMNH 98420

FMNH 98476

*Apogon pillionatus* 3 CAR ANSP 123818

ANSP 144339

ANSP 149005

*Apogon planifrons* 7 CAR FMNH 98122

FMNH 98562

FMNH 98600

FMNH 98605

LACM 8940-15

*Apogon pseudomaculatus* 6 CAR FMNH 64209

FMNH 64214

FMNH 65175

FMNH 65193

LACM 2457

*Apogon quadrisquamatus* 4 CAR ANSP 97026

*Apogon retrosella* 7 EPAC LACM 6963-9

LACM 6981-8

*Apogon townsendi* 11 CAR FMNH 97254

FMNH 98065

FMNH 98481

LACM 4563

*Astrapogon alutus* 2 CAR LACM 5374

*Astrapogon puncticulatus* 7 CAR FMNH 98111

FMNH 98187

FMNH 98195

FMNH 98205

FMNH 98217

*Astrapogon stellatus* 3 CAR LACM 5378

LACM 6717-1

*Paroncheilus affinis* 4 CAR ANSP 144100

ANSP 144192

*Phaeoptyx conklini* 9 CAR LACM 5379

LACM 5380

LACM 5389

*Phaeoptyx pigmentaria* 9 CAR LACM 2556

LACM 5397

LACM 31573-7

LACM 36218-7

*Phaeoptyx xenus* 8 CAR FMNH 82586

FMNH 98063

FMNH 98075

FMNH 98121

FMNH 98434

LACM 6834-1

*Zapogon evermanni* 4 CAR ANSP 144099

ANSP144100

FMNH 118475

FMNH 119001
